# Using satellites to uncover large methane emissions from landfills

**DOI:** 10.1126/sciadv.abn9683

**Published:** 2022-08-10

**Authors:** Joannes D. Maasakkers, Daniel J. Varon, Aldís Elfarsdóttir, Jason McKeever, Dylan Jervis, Gourav Mahapatra, Sudhanshu Pandey, Alba Lorente, Tobias Borsdorff, Lodewijck R. Foorthuis, Berend J. Schuit, Paul Tol, Tim A. van Kempen, Richard van Hees, Ilse Aben

**Affiliations:** ^1^ SRON Netherlands Institute for Space Research, Leiden, Netherlands.; ^2^ Harvard University, Cambridge, MA, USA.; ^3^ GHGSat Inc., Montréal, Quebec, Canada.

## Abstract

As atmospheric methane concentrations increase at record pace, it is critical to identify individual emission sources with high potential for mitigation. Here, we leverage the synergy between satellite instruments with different spatiotemporal coverage and resolution to detect and quantify emissions from individual landfills. We use the global surveying Tropospheric Monitoring Instrument (TROPOMI) to identify large emission hot spots and then zoom in with high-resolution target-mode observations from the GHGSat instrument suite to identify the responsible facilities and characterize their emissions. Using this approach, we detect and analyze strongly emitting landfills (3 to 29 t hour^−1^) in Buenos Aires, Delhi, Lahore, and Mumbai. Using TROPOMI data in an inversion, we find that city-level emissions are 1.4 to 2.6 times larger than reported in commonly used emission inventories and that the landfills contribute 6 to 50% of those emissions. Our work demonstrates how complementary satellites enable global detection, identification, and monitoring of methane superemitters at the facility level.

## Teaser

Landfill methane emissions with high potential for mitigation can be detected and quantified using a combination of satellites.

## INTRODUCTION

Reducing methane emissions is a priority for curbing climate change (*1*–*4*). With global methane concentrations increasing at record pace (*5*), identifying sources with high potential for mitigation is a crucial first step. A small number of anomalously strong point sources (“superemitters”) make up a disproportionately large fraction of total emissions and can often be readily mitigated (*3*, *6*, *7*). Satellites have the ability to observe atmospheric methane concentrations around the world. They can be used to detect and quantify strong point sources and characterize emissions at regional and national scales for comparison with reported emissions (*8*). Here, we leverage synergies between satellite instruments with disparate spatial resolution and coverage to detect strong urban methane hot spots, identify major sources responsible for the hot spots, and characterize their facility-level emissions.

Emissions from the oil and gas sector have received considerable attention (*9*–*15*), but there are also major opportunities for emission mitigation in the waste sector, which accounts for roughly 18% of global anthropogenic emissions (*16*). Solid waste emissions are caused by the anaerobic decay of organic material in landfills. Large historic methane emission reductions reported to the United Nations Framework on Climate Change (UNFCCC) by Annex-I countries have been related to landfills. Reported solid waste emissions in the United States fell by 38% between 1990 and 2018, and emissions in the European Union were nearly halved over the same time period (*17*). However, landfilled waste is expected to grow at more than double the rate of population growth between now and 2050, mainly driven by countries in the tropics (*18*). As a result, global municipal solid waste methane emissions could nearly double to 60 Tg a^−1^ by 2050 (*19*). Conversely, these emissions could be reduced to 11 Tg a^−1^ using technically feasible reduction strategies including active landfill covers, energy recovery, and omitting organic waste from landfills (*19*, *20*). In this study, we use a multisatellite observing framework to identify, characterize, and monitor four high-emitting landfills across the globe, including the ability to track emission mitigation measures.

Launched in October 2017, the Tropospheric Monitoring Instrument (TROPOMI) on the Sentinel-5P satellite provides daily global coverage of atmospheric methane concentrations at a spatial resolution of up to 5.5 × 7 km^2^ (*21*, *22*). These data can be used to detect and quantify large emission events (*11*, *12*) with a detection threshold of ∼5 t hour^−1^ under ideal circumstances (*8*) and regional emissions (*14*, *15*), but the spatial resolution is insufficient to unambiguously pinpoint emissions from all but the strongest and most isolated methane point sources. Meanwhile, target-mode instruments such as GHGSat-D, GHGSat-C1, and GHGSat-C2 (*23*, *24*) only observe limited spatial domains (∼10 × 15 km^2^) but do so at fine pixel resolution of up to approximately 25 × 25 m^2^ (*25*). When targeting locations with enhanced methane concentrations detected by TROPOMI, the GHGSat satellites can be used to identify individual sources and quantify their emissions. With its global coverage, TROPOMI can therefore guide (“tip and cue”) target-mode observations and provide a powerful tool to identify strong point sources when combined with instruments like GHGSat. Because the GHGSat field of view is similar to the footprint of a single TROPOMI observation, TROPOMI data from multiple days need to be analyzed alongside wind information to determine the target locations with sufficient spatial precision.

## RESULTS

**Fig. 1.**
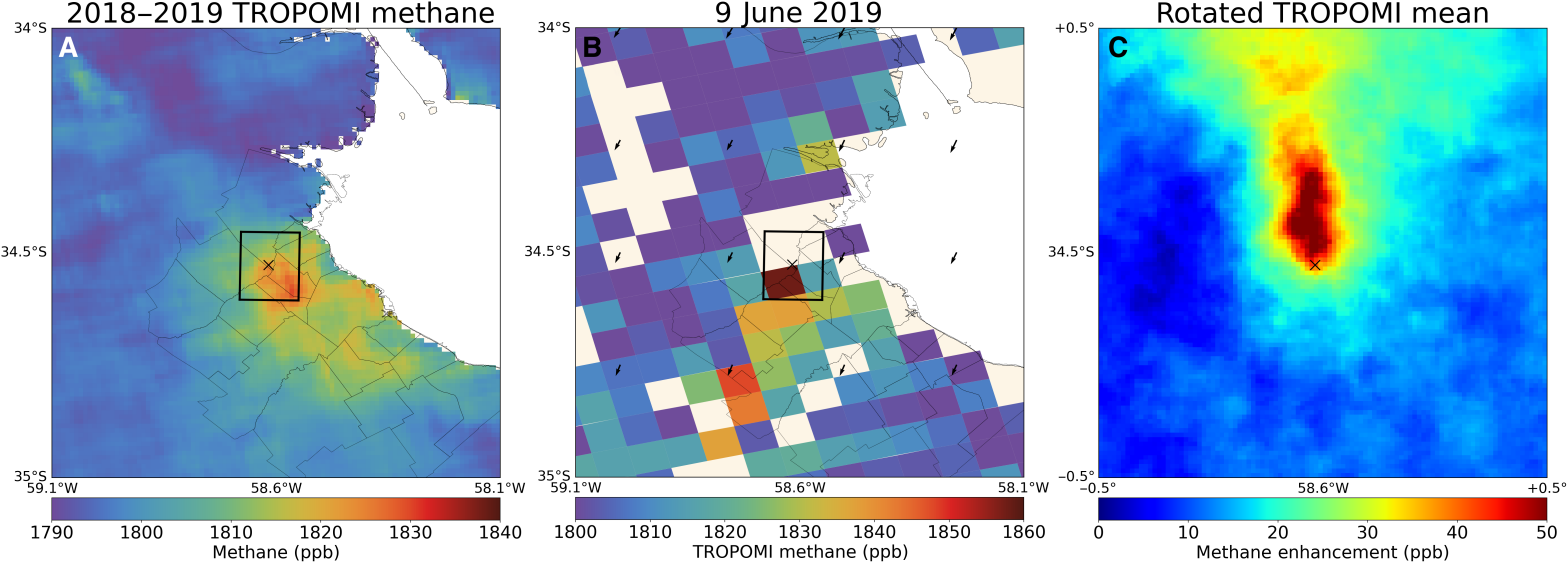
TROPOMI observations over Buenos Aires (Argentina). (**A**) Mean 2018–2019 TROPOMI methane concentrations oversampled (i.e., accounting for the full footprint of the observation) on a 0.01° grid. The Norte III landfill is indicated by the black cross; also shown are a GHGSat window centered on the TROPOMI-derived target (thick lines) and the Greater Buenos Aires municipalities [thin lines (*60*)]. (**B**) A single TROPOMI overpass on 9 June 2019 exhibiting a methane plume downwind of Buenos Aires with wind arrows representing ERA5 10-m winds (*28*). (**C**) The 2018–2019 wind-rotated average giving a clear (north-oriented) plume signal indicating a concentrated source.

We use long-term averages of TROPOMI methane data (*22*) to identify locations with persistently enhanced methane concentrations. Some of these locations have been shown to align with areas of known oil/gas production (*10*, *26*) or coal mining (*27*), but we also frequently find large enhancements over urban areas, such as Buenos Aires (Argentina; Fig. 1A). To identify the best target point for GHGSat within these (often spread-out) hot spots, we use a wind-rotation technique. For a potential target point, we rotate the data on individual days (e.g., Fig. 1B) based on the wind direction at 10 m from the ERA5 reanalysis meteorological fields (*28*), such that the wind vector is always oriented to the north. Where the target aligns with the methane source, the downwind concentrations are consistently enhanced compared to those upwind, resulting in a northward-oriented plume signal in the oversampled average of the rotated data (Fig. 1C) (*29*–*32*). By evaluating wind rotations for a dense grid of rotation points covering the area of interest, we can find which rotated average shows the largest downwind enhancement and thus pinpoint the source’s location to within a few kilometers (see Materials and Methods). We apply this wind-rotation method to 2018–2019 TROPOMI data over Buenos Aires and find the optimal target (34.53°S, 58.60°W) within 2 km of the Norte III landfill. We follow the same procedure for Delhi (India), Lahore (Pakistan), and Mumbai (India), where we also identify landfills as optimal targets for GHGSat observations (see Materials and Methods).

**Fig. 2.**
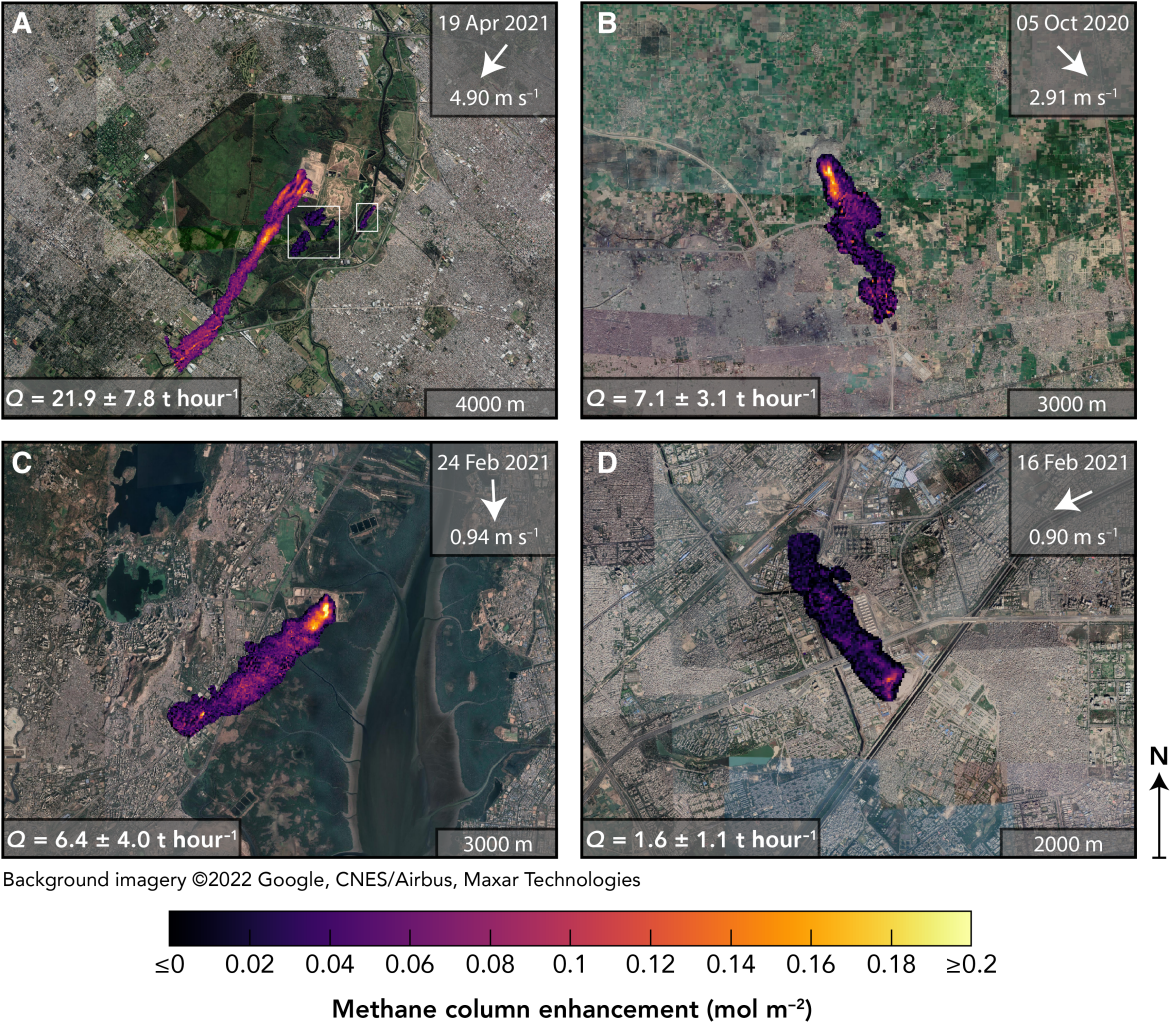
Methane plumes observed by GHGSat-C1/C2. (**A**) Norte III (Buenos Aires, Argentina), (**B**) Lakhodair (Lahore, Pakistan), (**C**) Kanjurmarg (Mumbai, India), and (**D**) Ghazipur (Delhi, India) landfills in 2020 and 2021. Concentrations are plotted over aerial imagery. Wind directions are from GEOS-FP (*52*), and emission quantifications (Materials and Methods) are shown in the legend. The leftmost plume in (A) is truncated at the edge of the viewing domain and quantified at 19.1 ± 6.7 t hour^−1^; the other plume from the landfill and plume across the river (circumscribed by the white boxes) are quantified at 2.7 ± 1.0 and 1.6 ± 0.6 t hour^−1^, respectively. The plume across the river is not incorporated in the estimate of the landfill’s total emissions.

Figure 2 shows a sample of typical methane plumes detected by GHGSat-C1/C2 from the Norte III (Buenos Aires), Lakhodair (Lahore), Kanjurmarg (Mumbai), and Ghazipur (Delhi) landfills. Plume shapes are generally consistent with the wind direction from the GEOS-FP meteorological reanalysis. To quantify the emissions, we use GEOS-FP wind speeds (the method is independent of the wind direction) in an integrated mass enhancement (IME) calculation (*33*, *34*). The IME method relates source rate *Q* to the total excess methane mass in the plume. Previous studies used large-eddy simulations (LESs) of methane point sources to calibrate the *Q* = *f*(IME) relationship. Here, we perform an area-source calibration that is more appropriate for application to landfills, where emissions may originate diffusely from a surface (see Materials and Methods). We estimate mean methane emission rates between 3 and 29 t hour^−1^ for the four landfills [Table 1; full time series including GHGSat-D observations starting in December 2019 (fig. S8) are shown in fig. S7]. Whenever there are clear-sky GHGSat-C1/C2 observations over the four sites, we detect emission plumes. Uncertainty in the estimated emissions (see Materials and Methods) and uncertainty in wind direction increase with decreasing wind speed (*35*), which can be seen from the mismatch between plume direction and wind vector for the 16 February 2021 observation of the Ghazipur landfill (Fig. 2D). The mean emissions that we find for the Ghazipur landfill are within the range of 1.4 to 3.3 t hour^−1^ found for 2015 using emission models (*36*).

**Table 1.** City-level and facility-level emissions for the four landfills quantified using TROPOMI and GHGSat observations respectively (t hr^–1^).

	**Buenos Aires**	**Delhi**	**Lahore**	**Mumbai**
City-level inventory*	22	28	25	17
City-level TROPOMI†	58 (55–64)	40 (38–45)	50 (47–54)	37 (28–40)
Facility-level GHGSat‡	28.6 (15.8–57.8)	2.6 (1.6–3.8)	6.4 (2.3–16.0)	9.8 (6.1–26.0)
Landfill contribution	50%	6%	13%	26%

*Inventory (bottom-up) estimates are the sum of 2012 oil/gas/coal emissions from Scarpelli *et al.* (*39*), other 2015 anthropogenic emissions from EDGAR v5 (*40*), and 2017 wetland emissions from WetCHARTS version 1.2.1 (*41*). Cities are taken as a 0.8° box centered on the population-weighted centroid of the city.

†TROPOMI-based estimates are the result of an inversion using 2020 data. Ranges give the range of the inversion ensemble (see Materials and Methods).

‡GHGSat estimates are based on the average of IME quantifications using GHGSat-C1/C2 data from 2020 to 2021. Ranges represent the spread of the individual quantifications. The Buenos Aires estimate includes estimates from the active and closed modules.

The fine resolution of GHGSat observations permits attribution of emissions to different sections of the landfills. While emissions from the Indian and Pakistani landfills appear widely distributed across the sites, emissions from the Norte III landfill originate mainly from the active module on the western side. This part of the landfill accounts for 87% of the detected emissions shown in Fig. 2 and only has intermediate covering, whereas the older eastern areas of the landfill were covered and closed in 2014 and 2018 using a 1.2-m cover with an active gas collection system. Emissions originate specifically from the two active surfaces on the northwest and southwest of the active module (fig. S11) that receive waste from the city and province of Buenos Aires, respectively. At the active surfaces, waste is continuously deposited, and the intermediate cover is relocated because of waste being added. Several vent wells have been installed as temporary mitigation tools in the active module, but the active surfaces provide the largest windows for landfill gas to escape. GHGSat-D observations from February 2020 show isolated plumes from the individual surfaces on different days (fig. S8). Because of the complicated nature of methane migration through the landfill, emissions are difficult to predict, and no measurements are taken on the ground to characterize them. The GHGSat imagery shown here demonstrates how satellites can add information at a spatial scale finer than that of inventory calculations.

Total methane generation reported by the Norte III landfill for 2019 is equivalent to 16.5 t hour^−1^ and is calculated on the basis of the UNFCCC methodology (*37*) incorporating landfill-specific information on the disposed waste, landfill architecture, methane fraction, and climate. Emissions are calculated per module of the landfill, and the methane generation estimate does not take into account the gas extraction for the closed modules, which should substantially decrease net emissions. Whereas total methane generation is close to the GHGSat emission estimates (which average 29 t hour^−1^ but 21 t hour^−1^ excluding the highest quantification as an outlier), emissions from the active module (which has an extraction system under construction) are reported at just 4.3 t hour^−1^ for 2019. The GHGSat observations (2020 to 2021) therefore indicate that emissions from the active surface may be underestimated, while emissions from the closed modules are much lower and not always detected as plumes. On the 2 days where individual closed-module plumes are detected, they account for only 8 to 13% of total emissions from the landfill. This shows that the covering and extraction system are largely successful and that emissions from the landfill could decrease quickly once the active surfaces are closed.

To put these emissions in context, we also quantify emissions from the surrounding urban areas using 2020 TROPOMI data. Emissions are estimated using the Weather Research and Forecasting chemical transport model [WRF-Chem (*38*)] at 3-km resolution, scaling inventory emissions (*39*–*41*) in a gridded Bayesian inversion to obtain the best match between simulated concentrations and TROPOMI observations (see Materials and Methods). The resulting urban emissions are given in Table 1. We find that commonly used emission inventories underestimate Buenos Aires’s 2020 urban emissions by a factor 2.6 and those of the other cities by factors of 1.4 to 2.2. On the basis of the mean of the GHGSat observations, the observed landfills are responsible for 6 to 50% of the city-wide emissions. In Buenos Aires and Mumbai, the individual landfills account for more than a quarter of total urban emissions. The Norte III landfill makes up about half of Argentina’s solid waste emissions (49 t hour^−1^, with 26 t hour^−1^ coming from managed landfills) reported to the UNFCCC for 2016 (*42*), which is not unexpected as the Buenos Aires province houses 40% of Argentina’s population (*43*). The Lakhodair landfill alone accounts for 10% of the 2015 UNFCCC-reported solid waste emissions for Pakistan (*44*), despite the Lahore district making up only 5% of the country’s population (*45*). This reflects a need to refine the magnitude and spatial representation of landfill and urban emissions in global inventory databases.

## DISCUSSION

The complementarity of TROPOMI and GHGSat provides a powerful tool to detect, locate, and quantify emissions from strong methane point sources around the world. Detections can be used to inform operators and regulators and promote action on cost-effective methane emission reduction measures. After identification of the emitting facility, continued observation allows monitoring of emissions and evaluation of mitigation measures. The hybrid methodology demonstrated here can also be applied with the successors of TROPOMI (e.g., Sentinel-5) and be used to guide target-mode hyperspectral instruments [e.g., the Precursor and Application Mission (PRISMA) (*12*, *46*) and the Environmental Mapping and Analysis Program (EnMAP) (*47*)] or inspection of imagery from global-surveying high-resolution multispectral instruments [e.g., Sentinel-2 and Landsat (*26*, *48*)] and be supplemented with future intermediate-resolution data from instruments such as MethaneSAT (*49*). Combining these diverse data streams enables global identification of strong methane sources followed by facility-level monitoring necessary to reduce emissions in the short term, improve emission inventories for climate policy, guide ground-based measurement campaigns to better understand emissions, and support regulatory enforcement.

## MATERIALS AND METHODS

### TROPOMI data and source localization

TROPOMI is a pushbroom spectrometer that was launched aboard the Sentinel-5P satellite in October 2017 (*21*, *22*). It retrieves methane with daily global coverage from the 2305- to 2385-nm shortwave infrared (SWIR) band and the 757- to 774-nm near-infrared band with 5.5 × 7 km^2^ resolution at nadir and a swath width of ∼2600 km at an overpass time of around 13:30 local time.

We use the TROPOMI methane product described by Lorente *et al.* (*22*) that shows good agreement [−3.4–parts per billion (ppb) average bias with 5.6-ppb station-to-station variability] with the Total Carbon Column Observing Network (*50*). For the source localization and the TROPOMI-based emission quantification, we use albedo-bias corrected data over land, filtered to include only measurements with the following: methane precision, <10 ppb; SWIR cloud fraction, <0.02; SWIR aerosol optical depth, <0.13 (0.10 for the inversion); and SWIR albedo, >0.02.

To identify regions of interest for closer inspection with GHGSat, we oversample 2018–2019 TROPOMI data at 0.01° × 0.01° resolution following Zhu *et al.* (*30*). In the oversampling approach, the full spatial footprint of the observation is taken into account by attributing the observed value to grid cells weighted by the spatial overlap of the observation with those grid cells. For a region to be of interest, we filter on the basis of the enhancement (defined as the difference between the TROPOMI retrieval and the a priori column used in the retrieval), require sufficient coverage at the considered grid cell and surrounding grid cells to filter anomalous values at the edges of the TROPOMI coverage, and require limited local correlation with SWIR albedo, SWIR aerosol optical depth, and coverage (fig. S1). Multiple regions of interest result, related to various emission sources, but here we focus on the Buenos Aires (Argentina), Delhi (India), Lahore (Pakistan), and Mumbai (India) urban areas.

After identifying a location of interest, we use a wind-rotation technique (*29*, *32*) to pinpoint the potential target location with sufficient precision so the source will fall within GHGSat’s ∼10 × 15 km^2^ field of view when targeting this location. The rationale behind this method is that simply averaging the TROPOMI data will result in smearing of signal due to varying wind directions on different days. Rotating TROPOMI methane enhancements around a source location such that the wind is always pointing north will lead to aligning plumes on different days. This is the result of downwind concentrations always being larger than upwind concentrations at a source location. The rotated 2018–2019 data are then oversampled at 0.01° resolution, resulting in an average downwind “plume-like” signal. We perform this wind rotation for a full grid of 13 × 13 points across the region of interest, first distanced at 0.05° (shown for Buenos Aires in fig. S2) and subsequently at 0.01° (fig. S3) to determine which location has the largest downwind enhancement and hence is the most likely location of the source. Wind data come from a spatial spline interpolation (at the target location) of the hourly 10-m wind field closest in time to the TROPOMI overpass from the 0.25° × 0.25° ERA5 reanalysis product (*28*).

To analyze which rotated image is centered at the most likely source location, we compute several metrics based on the oversampled averages (fig. S4). These metrics are the mean enhancement in a 0.25° × 0.05° box downwind of the source, the difference between that enhancement and the enhancement in the 0.25° × 0.05° box upwind, and the maximum concentration downwind of the source. We look at the agreement between these metrics to estimate the most likely source location, usually best represented by the mean downwind concentration. For Buenos Aires, the optimized location is 34.53°S, 58.60°W ± 0.01°, and all optimized locations are given in table S1. The reported uncertainty of estimated location is based on the absolute distance between the two most-separated points for which the metrics peak. Where necessary, locations can be fine-tuned using external information from visual imagery or emission databases to ensure that the most likely (or maximum number of) emission targets are within the GHGSat field of view. This is necessary in Lahore, for example, where there is a large diffuse background emission from the city. Mean TROPOMI concentrations and rotated averages centered at the landfills for the three other targets are shown in fig. S5.

### GHGSat data, emission quantification, and uncertainty

GHGSat satellite instruments are wide-angle imaging Fabry-Perot spectrometers that retrieve atmospheric methane columns by solar backscatter in the 1630- to 1675-nm SWIR spectral range. The demonstration instrument GHGSat-D was launched in June 2016 and observes at around 10:00 local time, with a return time of 2 weeks. It has a targeted field of view of ∼10 × 10 km^2^ with an effective pixel resolution of 50 × 50 m^2^ and is described in detail by Jervis *et al.* (*23*). Follow-up instruments GHGSat-C1 and GHGSat-C2 were launched in 2020 and 2021 with an improved detection limit, effective pixel resolution of approximately 25 × 25 m^2^, and a targeted field of view greater than ∼10 × 15 km^2^ (*24*). The latest instruments achieve a median retrieval precision of 1.5% of background across all scenes and observing conditions. They can detect point sources down to 100 kg hour^−1^ as determined using controlled releases (*51*).

We use the IME method (*7*, *33*, *34*) calibrated with LESs to quantify emissions with GHGSat observations. Varon *et al.* (*34*) calibrated IME source-rate retrievals using LESs of methane plumes originating from point sources. Here, we adopt the same calibration approach but with a uniform square area source to estimate source rates for the landfill plumes detected by GHGSat (Fig. 2). The IME method relates the source rate *Q* to the total methane mass (IME) of the plume

$$Q=\frac{U_{\text{eff}}\text{IME}}{L}$$ (1)

where *U*_eff_ = *f*(*U*_10_) is an effective wind speed that can be expressed as a function of the local 10-m wind speed *U*_10_, and *L* is a plume length scale commonly defined as the square root of the area (*A*) of the detectable plume: $L=\sqrt{A}$. The plume area is calculated from a binary plume mask that distinguishes plume pixels from background pixels. We define the mask in the same way as Varon *et al.* (*10*) did, by applying a threshold to the retrieved columns and smoothing the resulting mask. We use 10-m wind speeds from GEOS-FP (*52*).

Calibrating the IME source-rate retrieval involves characterizing the effective wind speed for a set of measurement conditions, either as a function of *U*_10_ (*34*) or based on the shape of the observed plume (*53*). Here, we use the former approach and compute source rates by mapping *U*_10_ as reported in the GEOS-FP meteorological database to an effective wind speed. We perform five 3-hour-long simulations of a 275 × 275–m^2^ area source representing the active surface of a landfill, using a variety of meteorological conditions [a range of mixed layer depths (500 to 2000 m) and sensible heat fluxes (100 to 300 W m^−2^)]. Our model setup uses the WRF v3.8 default LES case (*54*, *55*) as described by Varon *et al.* (*48*), but with an area source rather than a point source. In the five-simulation ensemble, each simulation covers 3 hours and a 9 × 9 × 2.4–km^3^ domain with 25-m horizontal and 15-m vertical resolution. The first hour of each simulation is used to spin up turbulence, and data from the last 2 hours are used to determine the relationship between *U*_10_ and *U*_eff_ in the IME method. In parallel, we also use the point-source simulation ensemble from Varon *et al.* (*48*).

Drawing snapshots from these two LES ensembles in 30-s intervals, we obtain 1200 samples each of area- and point-source plumes. We scale the snapshots to reflect random source rates in the range of 2 to 30 t hour^−1^. We integrate the snapshots vertically and add synthetic measurement noise drawn from a normal distribution with mean zero and SD 5% of a 1875-ppb methane background. This noise level (retrieval precision) is determined from the GHGSat-D, GHGSat-C1, and GHGSat-C2 retrieval fields for the four landfills, as the average SD of nonplume methane enhancements across all the observed scenes. In this manner, we obtain 2400 GHGSat pseudo-observations of point-source and area-source plumes. We then follow the methodology of Varon *et al.* (*34*) to derive effective wind speed functions from the two synthetic plume datasets.

Figure S6 shows the resulting effective wind speed functions for area sources and point sources. We find that first-degree polynomials capture the dependence well (0.78 < *R*^2^ < 0.86) in both cases. The linear fits for the two populations are similar, but the effective wind speed is generally higher and more variable for area sources than for point sources. This is because area-source plumes are more diffuse and tend to have lower enhancements than point-source plumes of similar source strength. Weaker enhancements for area-source plumes are counterbalanced by higher effective wind speeds to recover the known *Q* during calibration, and the reduced signal leads to higher uncertainty in the effective wind speed needed for each plume snapshot. Best-fit lines are computed by robust linear regression, which assigns less weight to outlier points, to mitigate the impact of marginally detectable LES plumes on the effective wind speed fit. For the same reason, fig. S6 excludes plumes with IME below the 10th percentile of each LES ensemble.

Varon *et al.* (*34*) found a similar range of effective and 10-m wind speeds for their LES methane point-source plumes, but a logarithmic dependence of *U*_eff_ on *U*_10_ rather than the linear dependence that is shown here. This may be due to differences in spatial resolution and/or meteorological settings between Varon *et al.*’s (*34*) LES ensemble and the ensembles used here. Source rate estimates using a linear or logarithmic fit are, however, similar for the range of wind speeds covered by the LES ensembles, where absolute differences are, on average, less than 6%. Larger deviations can occur under low (*U*_10_ < 1.5 m s^−1^) and high (*U*_10_ > 6 m s^−1^) wind conditions. We use the area-source calibration of fig. S6 to report the best estimates for the landfill plumes observed by GHGSat (Fig. 2) and the point-source calibration to estimate error from uncertainty in the source shape.

We estimate the uncertainty in our retrieved source rates similarly to Varon *et al.* (*10*), accounting for wind speed error, model error in the IME method, and error from measurement noise. We include an additional error term to account for uncertainty in the shape and spatial extent of the source. The emissions detected by GHGSat may originate from a combination of gas extraction wells, active working faces, gaps in the landfill cover, and other potential methane sources at the target landfills. The true spatial distribution of the emissions may therefore be highly complex, but our source quantification scheme assumes that emissions are distributed uniformly across a 275 × 275–m^2^ area. To estimate the resulting error, for each LES plume, we perform a separate source-rate retrieval calibrated with our point-source ensemble and compare the implied emission rate *Q*_p_ with the result *Q*_a_ from the area-source retrieval. We estimate the error from source shape uncertainty as the SD of the differences between *Q*_a_ and *Q*_p_ for each landfill site, which comes to <15% on average. We also calculated the emissions using ERA5 10-m wind fields. We find that there is a 9% low bias when using ERA5 and GEOS-FP, but the mean absolute relative error between using ERA5 and GEOS-FP is only 15 ± 13%, easily encompassed by the mean wind speed error of 52% used in our reported results. Last, since the measurement noise depends on both target scene and satellite instrument, we include an additional error of 10% for using a single *U*_eff_ calibration (assuming 5% noise) to retrieve source rates for all four scenes and three satellites. The alternative would be to use 12 separate calibrations, but we use 1 for simplicity and because, in practice, the error is small. Our additional 10% error is a conservative estimate from a comparison of *U*_eff_ functions calibrated with 5% versus 20% retrieval precision, which have a mean absolute difference of 8.7%. Combining all sources of error in quadrature, we find total uncertainties (1σ) of 30 to 79% for landfill emissions quantified with GHGSat.

### TROPOMI emission quantification and uncertainty

We use version 4.1 of the WRF model (*38*) to simulate 240 × 240–km^2^ domains around the four landfills at 3-km resolution from 1 January 2020 to 1 January 2021. The simulations use meteorological fields from the National Centre for Environmental Prediction (NCEP) (*56*) and initial and 6-hourly boundary conditions at 0.25^∘^ × 0.25^∘^ from the Copernicus Atmosphere Monitoring Service (CAMS) (*57*). Our simulations use the tropical suite of physics options as transport configuration and provide hourly output.

We use bottom-up oil/gas/coal emissions for 2012 from Scarpelli *et al.* (*39*), and the remaining anthropogenic emissions are 2015 emissions from EDGAR v5 (*40*). Wetland emissions (2017) come from WetCHARTs version 1.2.1 (*41*) mapped to high-resolution wetland maps (*58*). Bottom-up prior emissions for each of the urban areas are given in table S2.

To estimate mean 2020 emissions, simulation output for that year is sampled using the TROPOMI averaging kernels at the model time step closest to the TROPOMI overpass time. To reduce the impact of possible model errors, we aggregate the TROPOMI observations and their model equivalents to a daily 0.2° × 0.2° grid and use those aggregated data in a Bayesian inversion to optimize state vector $\hat{x}$

$$\hat{x}=x_{A}+S_{A}K^{T}{\left(KS_{A}K^{T}+S_{O}\right)}^{-1}(y-Kx_{A})$$ (2)

with posterior error covariance matrix $\hat{S}$

$$\hat{S}={\left(K^{T}{S_{O}}^{-1}K+{S_{A}}^{-1}\right)}^{-1}$$ (3)

and averaging kernel **A** giving the sensitivity of the solution to the true state

$$A=I-\hat{S}{S_{A}}^{-1}$$ (4)

where **x**_**A**_ is the prior state vector, **S**_**A**_ is the prior error covariance matrix, **K** is the Jacobian based on our WRF simulations including the aggregation, **S**_**O**_ is the observational error covariance matrix, **y** contains the aggregated TROPOMI observations, and **I** is the identity matrix. The posterior error covariance matrix can be normalized to the posterior error correlation matrix $\hat{S_{\text{cor}}}$ by dividing all terms by the square root of the associated diagonal terms.

Our 61-element state vector consists of monthly scaling factors on the CAMS boundary conditions to prevent bias in CAMS from compromising the inversion results and a 7 × 7 grid to scale the bottom-up emissions. When reporting results, the city-level emissions are calculated over 0. 8^∘^ × 0. 8^∘^ boxes centered on the population-weighted city centroids.

The prior error covariance matrix is assumed to be diagonal, and we assume errors of 50% for the different emissions and 10% for the CAMS boundary conditions. The observational error covariance is assumed to be diagonal as well, and the error on individual observations is estimated as the SD of the prior model-observation mismatch (17 ppb for Buenos Aires). If *n* observations fall within one 0.2° × 0.2° grid cell, we apply the central limit theorem ($∼\sqrt{n}$).

To estimate the uncertainty in our results, we generate an ensemble of sensitivity inversions by varying inputs and inversion assumptions. We report the range of these sensitivity inversions as the uncertainty on our emission estimates. The sensitivity inversions are as follows: (i and ii) increasing and decreasing the prior errors by a factor of 2; (iii and iv) using WRF model output sampled at the model time steps before and after the mean overpass time; (v and vi) performing the optimization with aggregation to 0.15° × 0.15° and 0.4° × 0.4° grid cells; (vii) offsetting both the latitude and longitude of the aggregation grid by 0.1°; (viii) using log-normal prior errors on the emissions following Maasakkers *et al.* (*59*); (ix) using a 1% prior error on the CAMS boundary conditions; (x) only using TROPOMI data with the highest quality flag (*QA* = 1); (xi) using the TROPOMI data without albedo correction; (xii) using the mean observational error for the aggregated observations instead of following the central limit theorem; and (xiii) optimizing just one annual CAMS boundary condition scaling factor.

Figure S9 shows the prior and posterior model simulations’ mismatches with the TROPOMI observations for 2020 over Buenos Aires. The prior model shows a large-scale underestimate across the model domain because of underestimated CAMS boundary conditions. This underestimate is corrected by scaling up the boundary conditions by, on average, 2.9% in the posterior model (center panel). Similar corrections (4 to 5% with some seasonality) are found for the other cities. The resulting posterior emissions, scaling factors, and the inversion’s averaging kernels for the emission grid are shown in fig. S10. The averaging kernels of the inversion show where the TROPOMI observations add information to the prior emissions. This is mainly the case for the considered urban area, where methane enhancements are seen in the TROPOMI data. Some bias unrelated to local emissions remains in the posterior model–observation mismatch. The resulting city-level emissions are given in Table 1.
