# Supplementary material for: Using satellites to uncover large methane emissions from landfills
Source: Sci Adv. 2022 Aug 10;8(31):eabn9683. doi: 10.1126/sciadv.abn9683 (PMC9365275; doi:10.1126/sciadv.abn9683)
Supplement: Supplementary file 1 — Supplements S1 to S4 Figs. S1 to S11 Tables S1 and S2 [file sciadv.abn9683_sm.pdf]

Science Advances
AAAS

# Supplementary Materials for

## Using satellites to uncover large methane emissions from landfills

Joannes D. Maasakkers *et al.*

Corresponding author: Joannes D. Maasakkers, j.d.maasakkers@sron.nl

*Sci. Adv.* **8**, eabn9683 (2022)
DOI: 10.1126/sciadv.abn9683

**This PDF file includes:**

Supplements S1 to S4
Figs. S1 to S11
Tables S1 and S2

# Supplementary materials

## Supplement 1: TROPOMI data and source localization

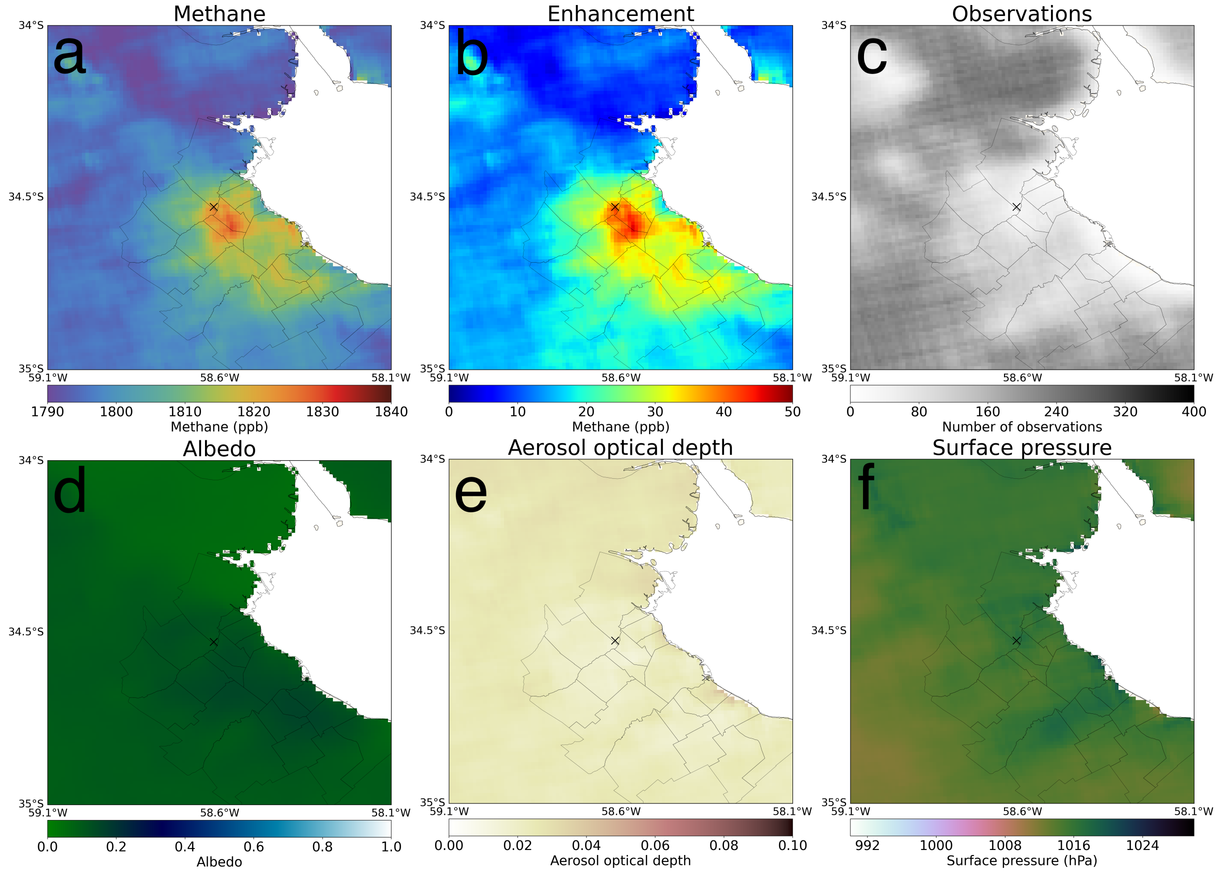


Figure S1: Oversampled 2018-2019 TROPOMI methane data over Buenos Aires (a) as well as supporting data used in the source localization procedure: methane enhancement (b), number of observations (c), SWIR surface albedo (d), SWIR aerosol optical thickness (e), and surface pressure (f). Also shown are the Greater Buenos Aires municipalities (thin lines (*60*)).

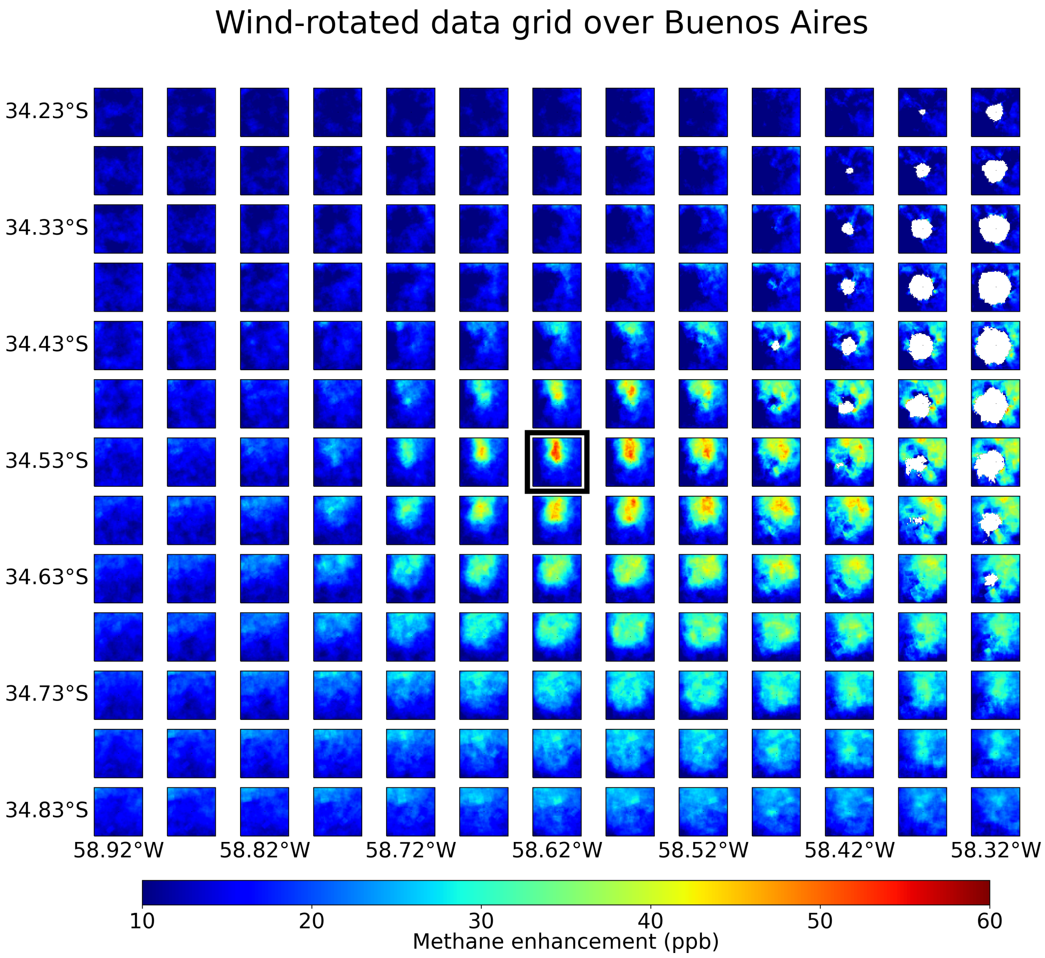


Figure S2: Rotated and oversampled 2018-2019 TROPOMI methane enhancements for a grid of rotation points separated by 0.05° spanning a region of 0.6° × 0.6° centered at the Norte III landfill in Buenos Aires. The image centered on the landfill is indicated by a black box. Each individual rotated image shows an area of 1° × 1°. Areas with fewer than ten TROPOMI observations are shown in white.

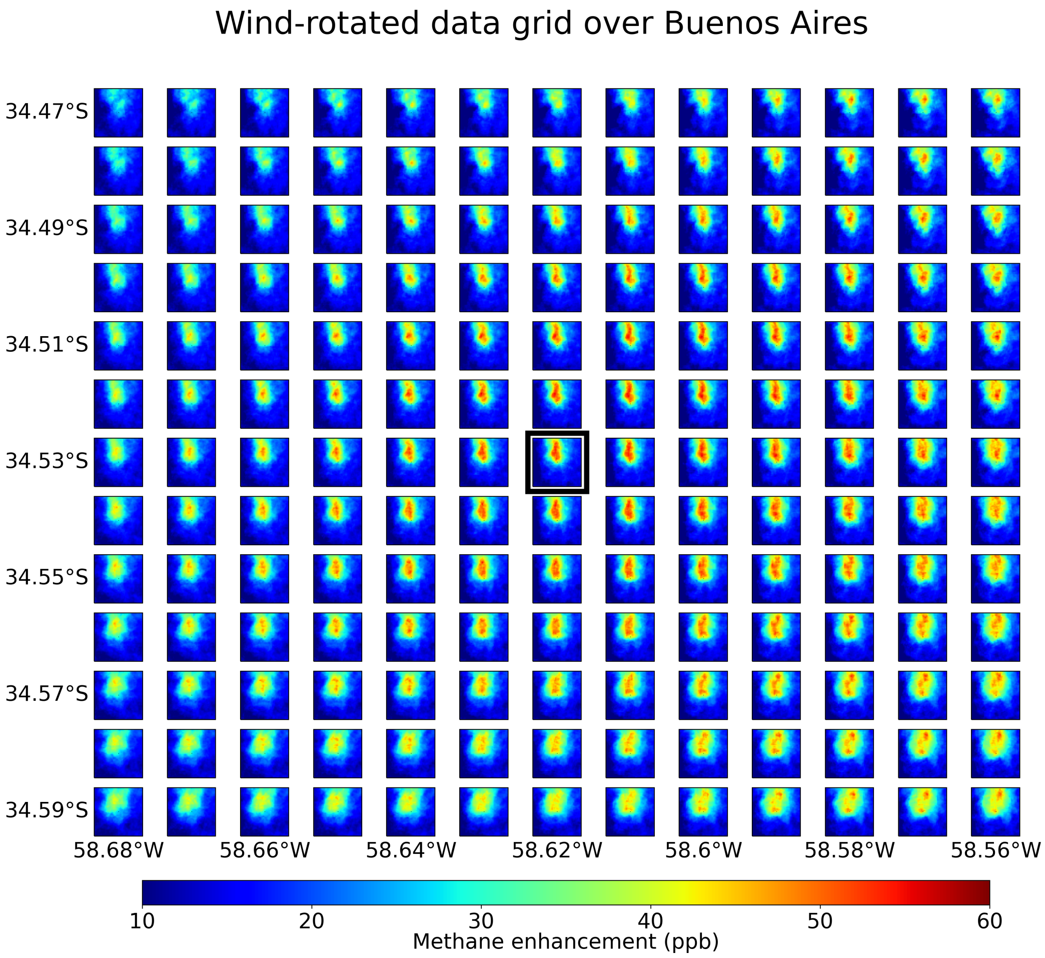


Figure S3: Rotated and oversampled 2018-2019 TROPOMI methane enhancements for a grid of rotation points separated by 0.01° grid covering an area of 0.12° × 0.12° centered at the Norte III landfill in Buenos Aires. The image centered on the landfill is indicated by a black box.

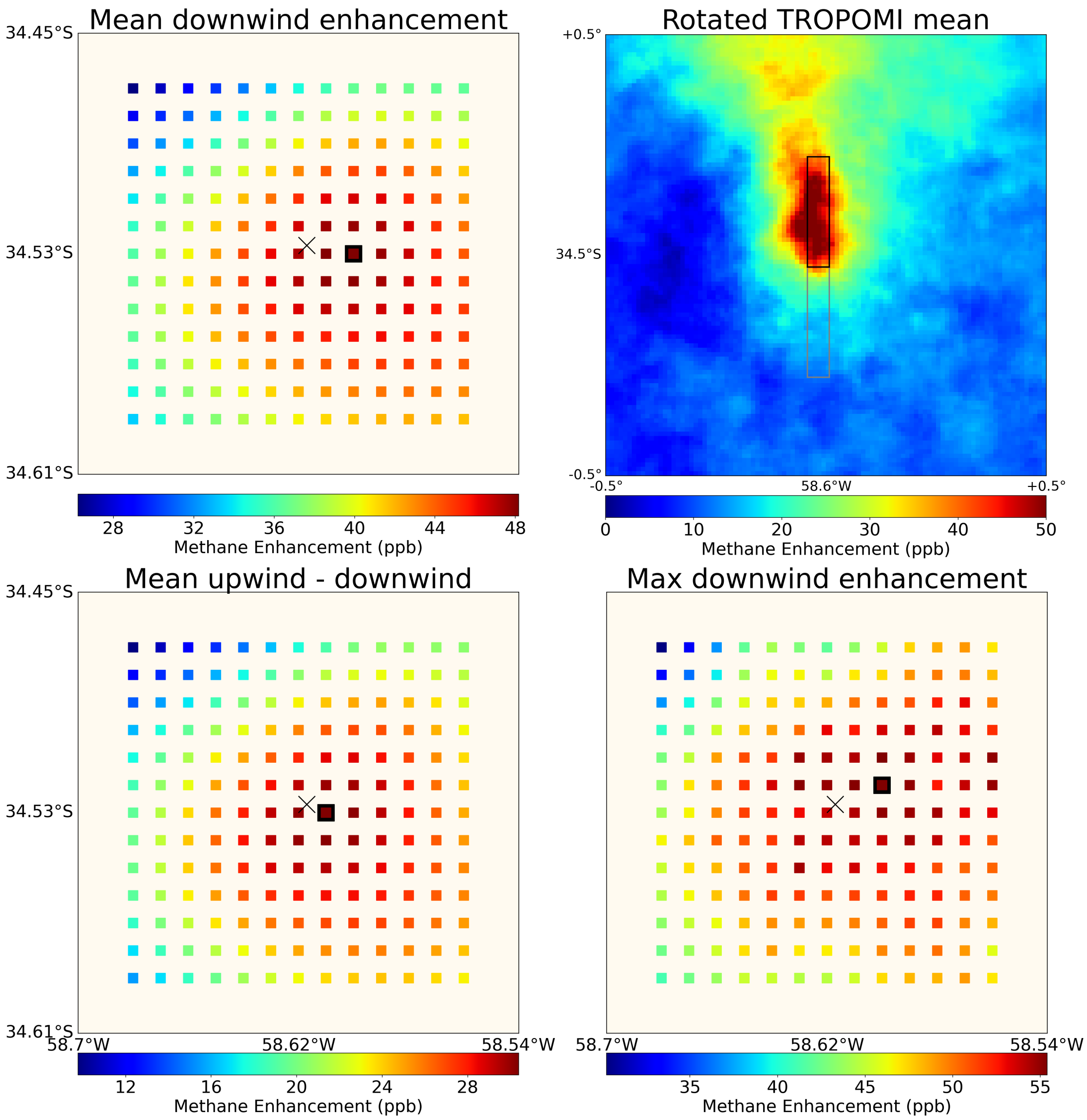


Figure S4: Metrics used to evaluate the grid of wind rotations. The top right figure shows the 0.25° × 0.05° downwind (black) and upwind (grey) boxes used in the computations on top of the rotated plume centered at the Norte III landfill. The computed values are the mean enhancements in the downwind boxes (top left); the difference between those enhancements and their upwind equivalents (bottom left); and the maximum enhancements downwind of the source (bottom right). The location of the landfill in the metric panels is marked by the black cross, the locations with the highest metric values are indicated with a black outline.

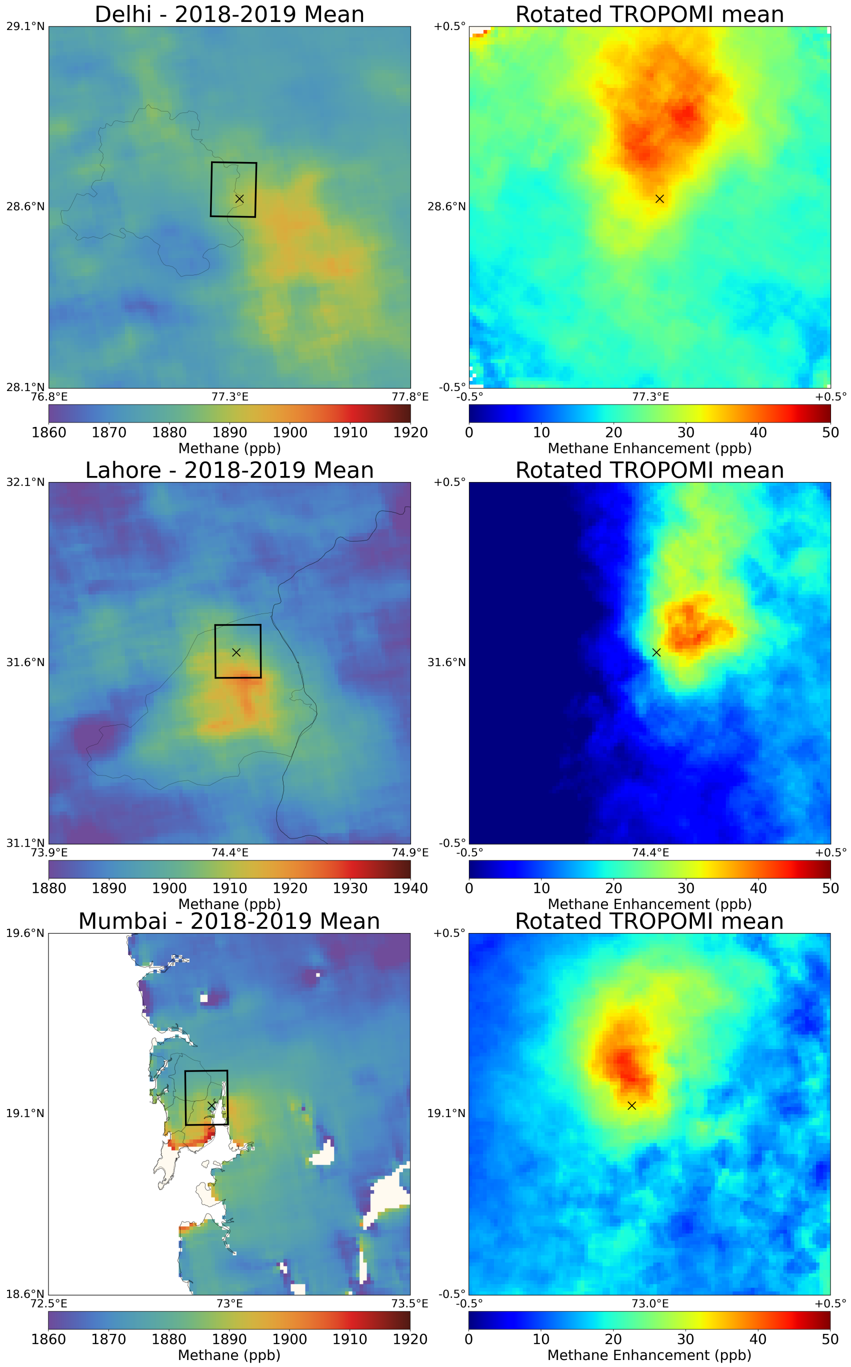


Figure S5: TROPOMI observations over Delhi (India), Lahore (Pakistan), and Mumbai (India). The left column shows mean 2018-2019 TROPOMI methane concentrations oversampled on a 0.1° grid. The targeted landfills are indicated by the black crosses, also shown are the targeted GHGSat windows and the respective urban areas (thin lines, for Mumbai including Suburban Mumbai (*60*)). The right column shows 2018-2019 wind-rotated averages centered on the landfills.

Table S1: Optimized target locations using the wind-rotation method on TROPOMI data

| | Optimized Location | Landfill Location |
|---|---|---|
| Buenos Aires | 34.53°S, 58.60°W ± 0.01° | 34.53°S, 58.62°W |
| Delhi | 28.63°N, 77.37°E ± 0.07° | 28.62°N, 77.33°E |
| Lahore | 31.54°N, 74.32°E ± 0.05° | 31.63°N, 74.42°E |
| Mumbai | 19.09°N, 72.89°E ± 0.03° | 19.12°N, 72.95°E |

# Supplement 2: GHGSat data, emission quantification, and uncertainty

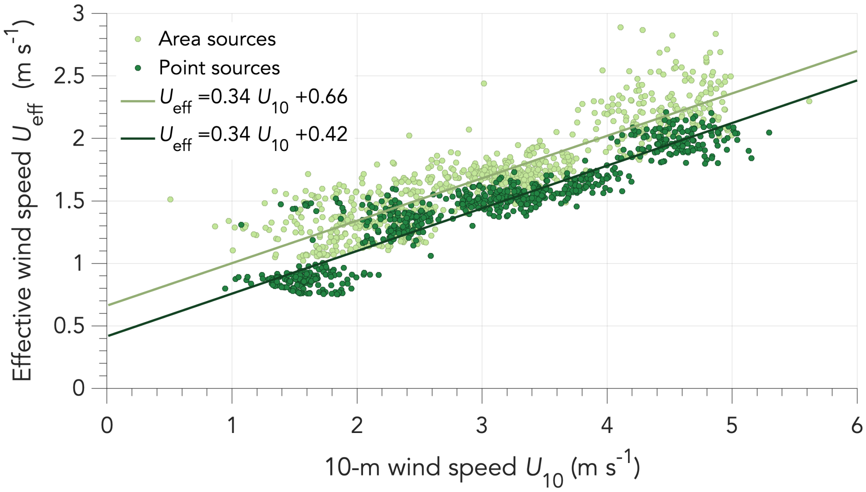


Figure S6: Effective wind speed fits for LES area sources and point sources in the IME method, and associated linear regressions.

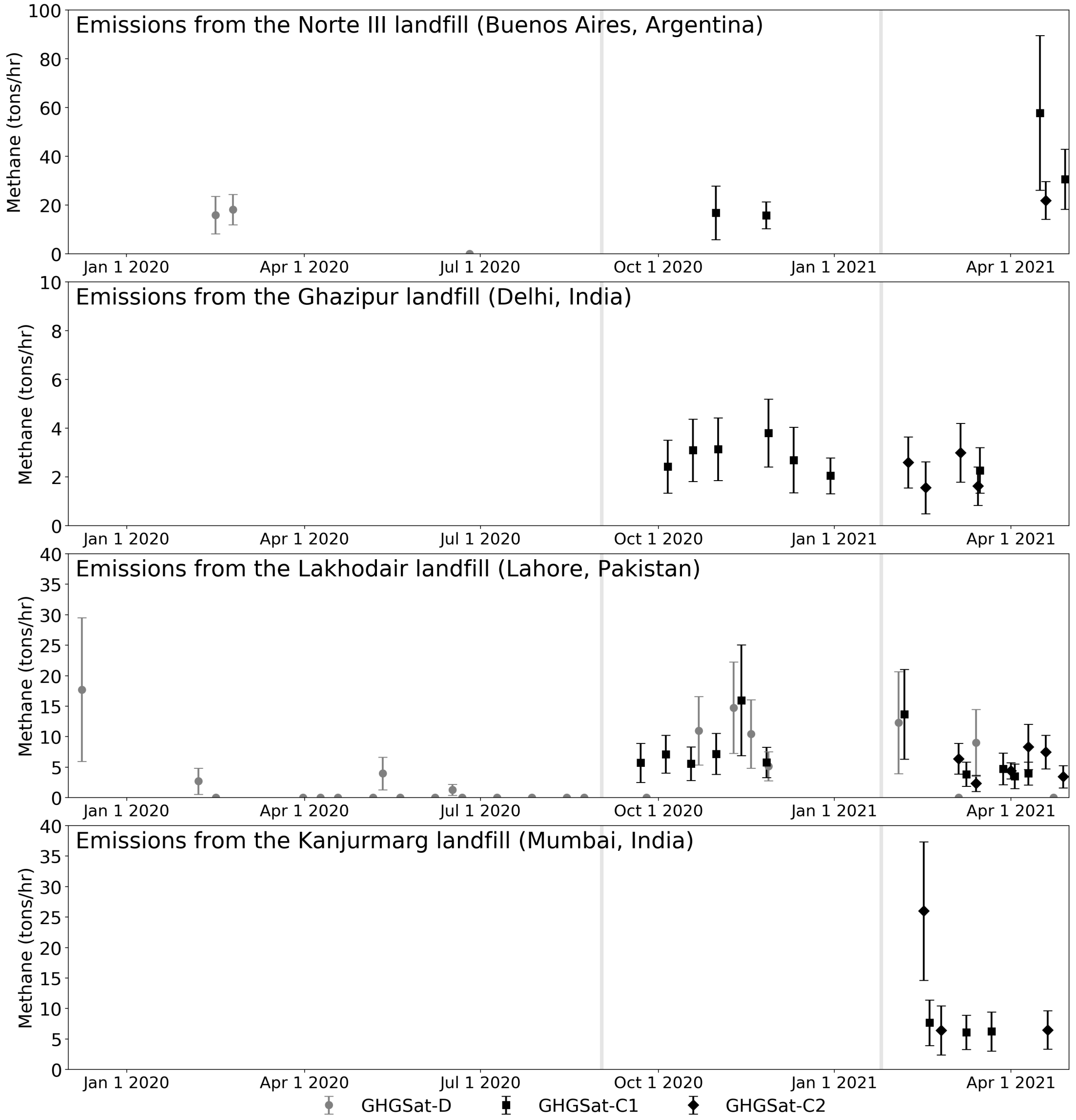


Figure S7: Time series of GHGSat-D/C1/C2 quantifications for the four landfills. Uncertainties are calculated as described in Supplement 2. Markers at 0 indicate clear scenes without any detected plumes, the grey background lines indicate the launch dates of GHGSat-C1/C2.

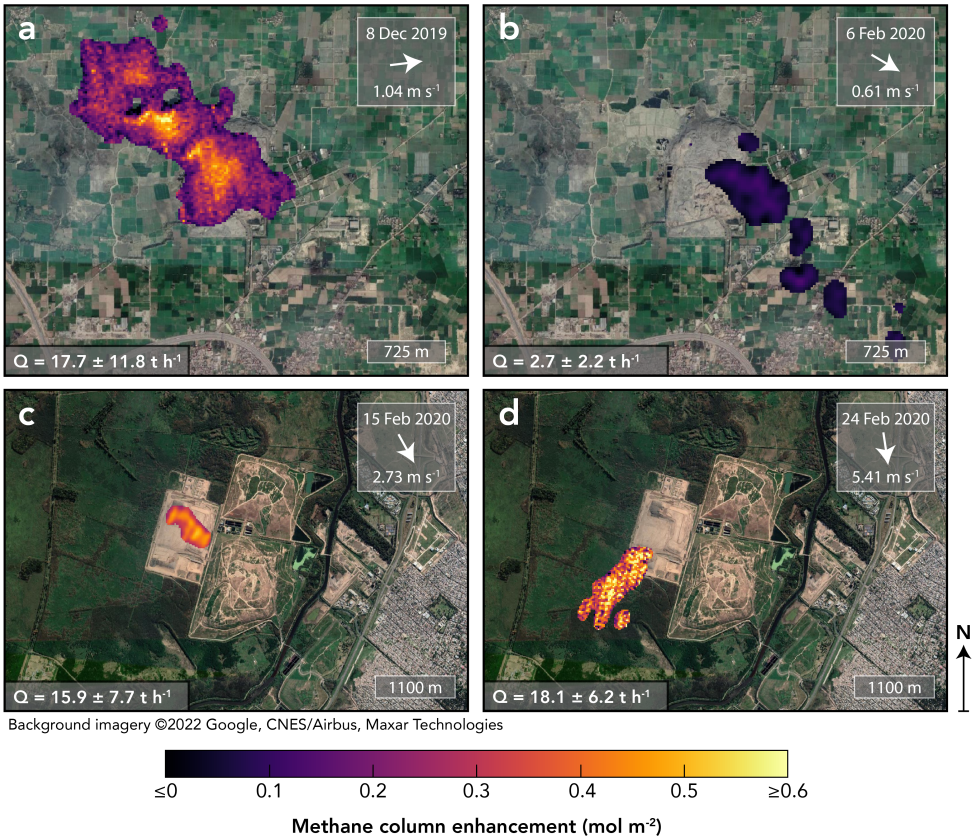


Figure S8: Methane plumes observed by GHGSat-D from the (a-b) Lakhodair (Lahore, Pakistan) and (c-d) Norte III (Buenos Aires, Argentina) landfills, in December 2019 and February 2020. Concentrations are plotted over high-resolution surface imagery. Wind directions are from GEOS-FP (*52*) and the emission quantifications are shown in the legend. The GEOS-FP wind directions are highly uncertain at the low wind speeds shown here. This uncertainty does not impact the quantification, because the IME method only uses the wind speed. The images show that even with the less sensitive GHGSat-D instrument, plumes can be detected from these landfills. The December 2019 plume over Lahore is - to our knowledge - the first landfill plume detected from space. The observations over Buenos Aires show that on two separate days, two different active faces of the landfill produce the largest methane signal.

# Supplement 3: TROPOMI emission quantification and uncertainty

Table S2: Prior emissions for the considered urban areas in t $hr^{-1}$

| Emissions[a] | Buenos Aires | Delhi | Lahore | Mumbai |
|---|---|---|---|---|
| Landfills | 3.8 | 7.2 | 1.6 | 5.6 |
| Wastewater treatment | 12.5 | 7.2 | 4.1 | 5.4 |
| Oil/Gas | 1.2 | 2.5 | 1.1 | 2.7 |
| Livestock | 1.0 | 8.5 | 14.6 | 1.8 |
| Rice | 0 | 0.6 | 2.4 | 0.3 |
| Coal | 0 | 0 | 0 | 0 |
| Other anthropogenic[b] | 0.6 | 1.8 | 1.2 | 0.9 |
| Wetlands | 3.2 | 0.1 | 0.1 | 0.1 |
| Total | 22.3 | 28.0 | 25.0 | 17.0 |

[a] Oil/gas/coal emissions for 2012 come from Scarpelli et al. (*39*); remaining anthropogenic emissions are 2015 emissions from EDGAR v5 (*40*); and 2017 wetland emissions (2017) are from WetCHARTs version 1.2.1 (*41*) mapped to high resolution wetland maps (*58*).

[b] Includes emissions from stationary/mobile combustion, industrial processes, fossil fuel fires, and solid waste incineration.

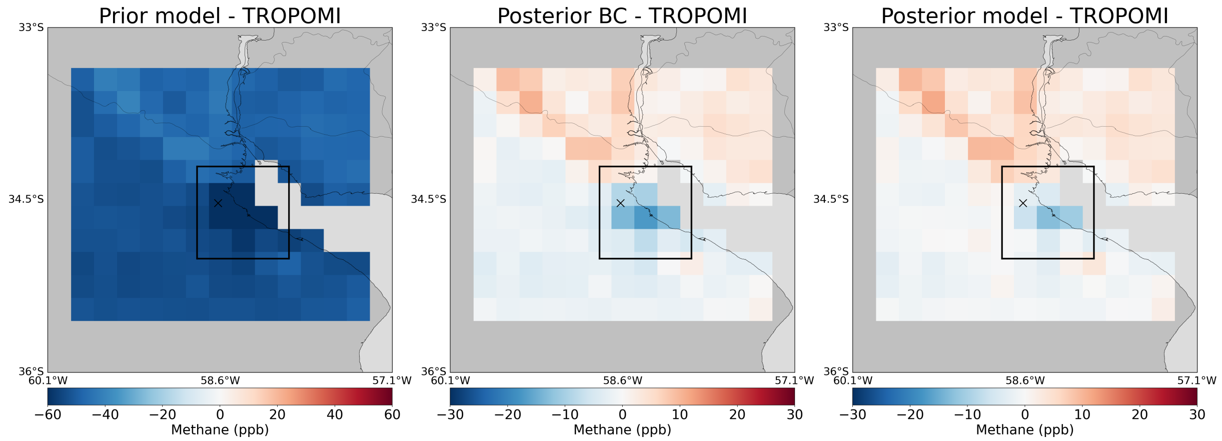


Figure S9: Model-observation mismatch using the prior (left), correcting the CAMS boundary conditions (center), and posterior (right) models for Buenos Aires compared to 2020 TROPOMI data. Differences are shown on the 0.2° × 0.2° aggregation grid used in the inversion, only grid cells with at least 20 days of observations are shown. The black boxes indicate the domain used to estimate urban emissions. The largest improvement can be seen around the city of Buenos Aires. Different color scales are used in the prior and posterior panels.

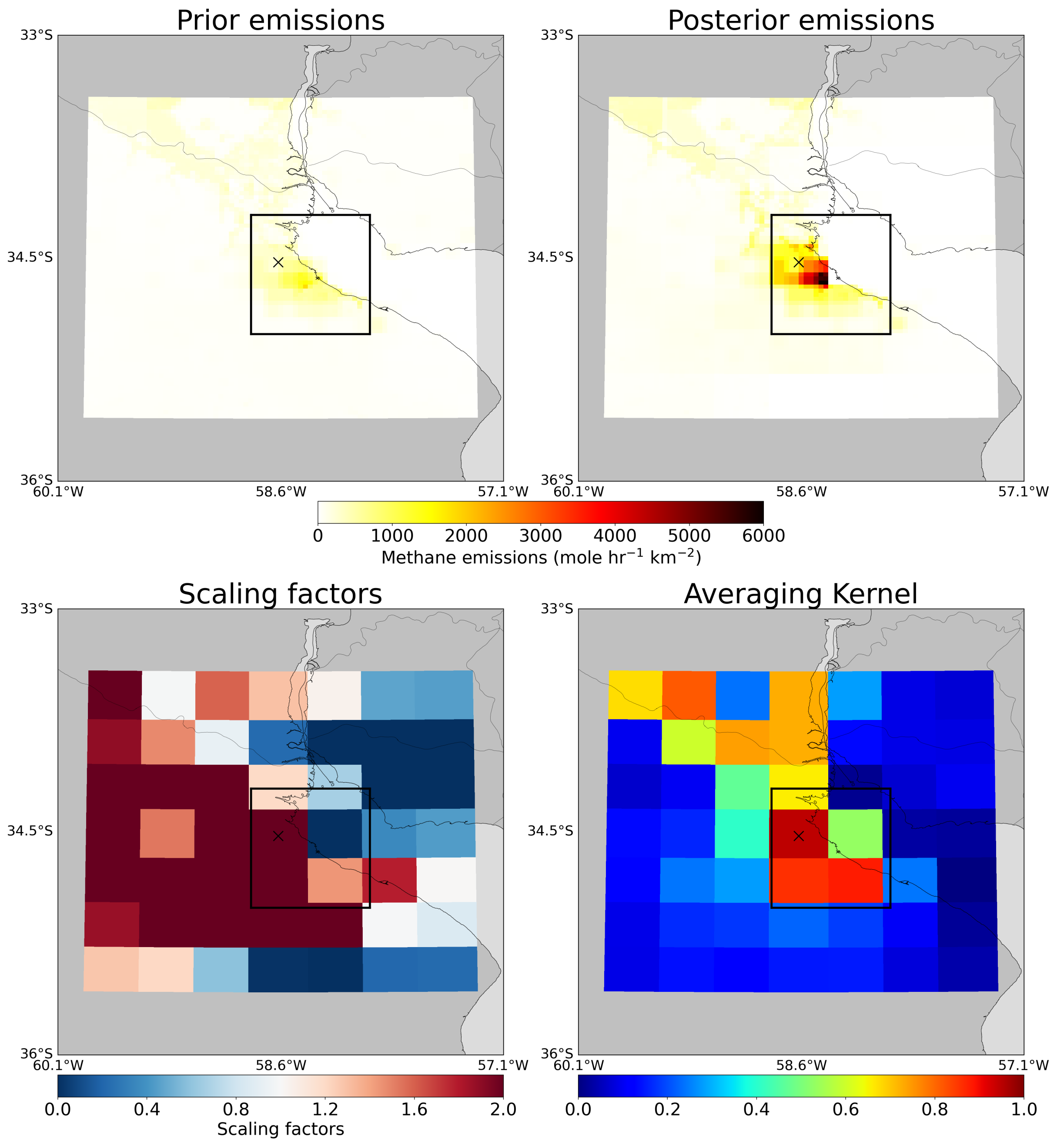


Figure S10: Prior (a) and posterior (b) emissions from the city-level TROPOMI inversion for Buenos Aires shown at the 3 × 3 km$^2$ WRF grid, the black box indicates the domain used to estimate urban emissions. Also shown are the resulting scaling factors (c) and the inversion's averaging kernels (d) at the resolution of the inversion.

## Supplement 4: Map of the Norte III landfill in Buenos Aires

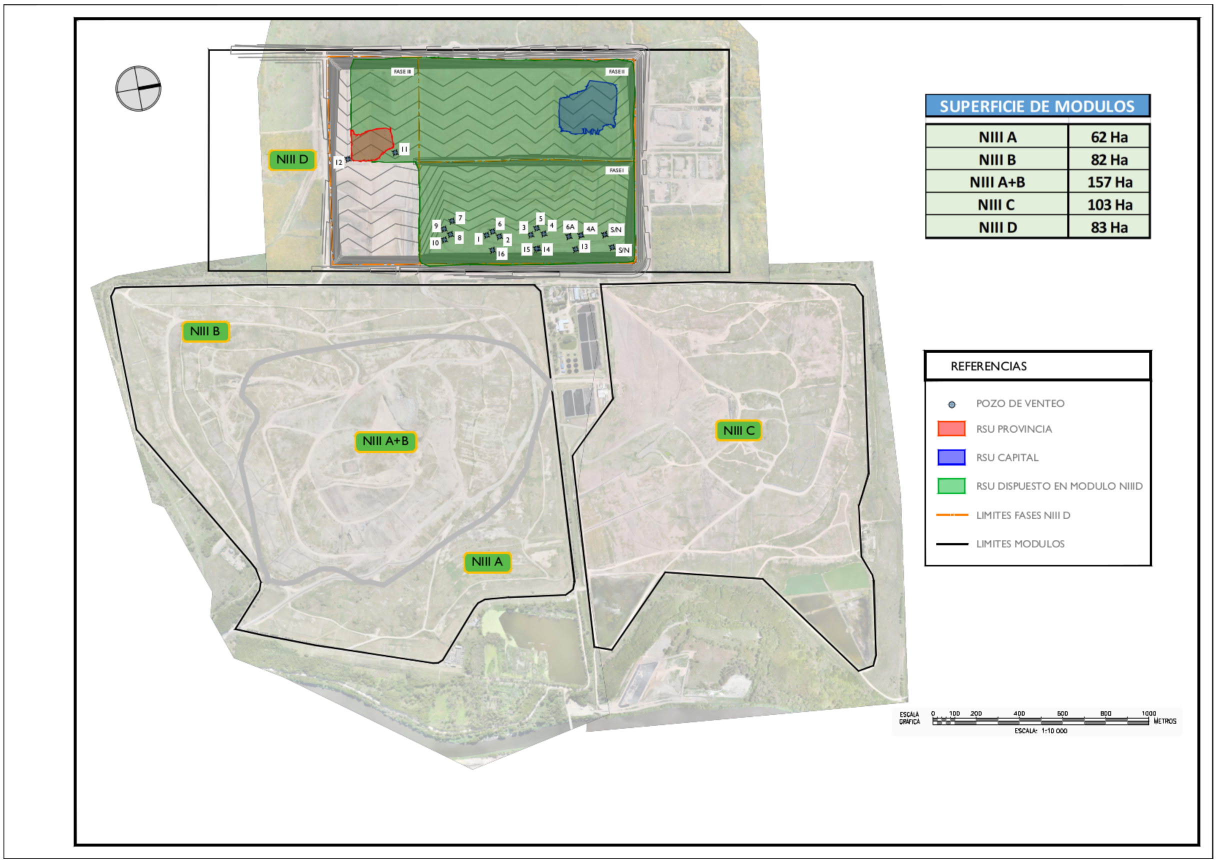


Figure S11: Overview of the Norte III landfill in Buenos Aires. The active module (D) is located on the western side (top of the figure) of the landfill and includes the provincial (red) and capital (blue) active surfaces. Boreholes are indicated by circles. The inactive modules A, B, and C are located on the eastern side of the landfill.
